# Association Between Anthropometric and Hematological Parameters and the Diagnosis of Intestinal Parasitosis in Low-Income Children

**DOI:** 10.3390/children11121416

**Published:** 2024-11-23

**Authors:** Bruno Freire, Alessandra Marques Sohn, Ricardo Rojas-Humpire, Salomon Huancahuire-Vega

**Affiliations:** 1 School of Medicine, Faculty of Health Sciences, Universidad Peruana Unión (UPeU), Lima 15464, Peru; brunofreire@upeu.edu.pe (B.F.); alessandrasohn@upeu.edu.pe (A.M.S.); ricardohumpire@upeu.edu.pe (R.R.-H.); 2 General Directorate of Research, Universidad Peruana Unión (UPeU), Lima 15464, Peru

**Keywords:** intestinal parasitosis, *Enterobius vermicularis*, pediatrics, diagnosis, anthropometry

## Abstract

Objective: This study aims to determine the association between anthropometric values and laboratory tests with parasitosis diagnosis and identify diagnostic models for parasitosis without relying on copro-parasitological examinations. Methods: Data were collected from 1894 children aged 0–14 who attended a medical center for low-income children in Lima, Peru, between 2021 and 2022. Anthropometric data (BMI, weight, height), laboratory data (red blood cells, hemoglobin, platelets, hematocrit, mean corpuscular volume, mean corpuscular hemoglobin, eosinophils), and parasitological examination results were analyzed. Prevalence ratios for the association between parasitosis and each anthropometric and laboratory variable were estimated using multivariable Poisson regression. Regression models were developed for each type of parasite found, and the diagnostic value was assessed using ROC curves. Results: A high prevalence of parasitosis was identified (41.9%), including *Blastocystis hominis* (29.1%), *Endolimax nana* (7.76%), *Entamoeba coli* (5.97%), *Giardia duodenalis* (6.44%), and *Enterobius vermicularis* (1.85%). It was found that the male sex (PRa 1.18), the age group of 2–5 years (PRa 4.83) and >5 years (PRa 4.59), the percentage of eosinophils (PRa 1.02 for every 1% increase), and height/age with −5 SD (PRa 1.34) were associated with a greater risk of parasitosis. Satisfactory values were only shown for diagnostic models *associating Enterobius vermicularis* and BMI, with a diagnostic value of 70.9% and 70.2% for a BMI < 12 and hematocrit > 29.8%, and BMI < 12 and hemoglobin < 10.6 g/L, respectively. Conclusions: Satisfactory diagnostic value models were only found for parasitosis by *Enterobius vermicularis*, suggesting the potential for reducing reliance on copro-parasitological exams in resource-limited settings.

## 1. Introduction

Parasitosis is a global health problem causing acute/chronic diarrhea, abdominal pain, malnutrition, and anemia [1,2]. Endoparasites in children are also associated with challenges in child development (height, weight, and cognitive development) [3,4].

In underdeveloped countries, the prevalence of parasites such as *Giardia duodenalis*, *Entamoeba histolytica*, and *Enterobius vermicularis* is higher in comparison to developed countries, the latter being the most common intestinal parasite in the world [5,6,7]. Parasitosis prevalence varies depending on the features of a certain population, since it is associated with the socioeconomic level, environmental sanitation, hygiene habits, and availability of treated water, among other factors [8]. Due to these relationships, parasitosis can be understood as an indirect index of poor socio-economic development. There is evidence of a lack of early diagnosis and effective treatment due to less access to diagnostic laboratories and health centers in developing countries, becoming a public health problem [9,10,11].

*Enterobius vermicularis*, also known as Pinworms, has a life cycle within the intestine of a single host (humans being the natural host), not needing an intermediate. It adheres to the intestinal mucosa, feeding on epithelial cells and commensal bacteria [7]. Contamination is predominantly fecal–oral, in addition to ingesting contaminated food and autoinfection [12]. Enterobiasis (*Enterobius vermicularis* infection) is one of the most common causes of malnutrition and growth retardation among children and is also associated with alterations in serum levels of iron, zinc, and magnesium [13,14]. These deficiencies could damage cellular, physiological, and enzymatic processes, which in the long term can explain alterations in the growth and development of the host. It is frequently asymptomatic; however, possible symptoms include anal itching that worsens at night, nocturnal enuresis, anal excoriation, insomnia, abdominal pain, anorexia, weight loss, nervousness, and difficulty concentrating, and can rarely debut with acute appendicitis [7,15,16].

Various risk factors associated with enterobiasis are school age, mother’s level of education, and nail size, as well as the habit of sucking fingers [10]. Paola Cociancic et al. and Juan Rojas et al. also point out risk factors related to housing and public services, including improvised material in houses, dirty floors, sharing a bed, lack of access to treated, clean water, lack of garbage disposal services, and living in a rural area [11,17]. Ahmed Al-Daoody suggests an association with family size, possibly due to direct transmission between humans without the need for an intermediate host [12]. Some studies suggest an association between the hemoglobin level and total proteins and enterobiasis, without a significant association with the levels of vitamin B12, folate, magnesium, phosphorus, and IgE [18,19].

Previous studies have shown that parasitic infections can have negative effects on the nutritional and hematological status of affected children. Nevertheless, the usefulness of anthropometric and hematological parameters as diagnostic markers has yet to be well established, hence the need to determine their diagnostic capacity in a low-income population and their combination with other diagnostic methods. Consequently, this work aims to identify these parameters’ diagnostic capacity for parasitosis in pediatric patients at a Health Center in Villa María del Triunfo, Lima, Peru, in order to reduce the delay in effective treatment in regions without the capacity to perform copro-parasitological testing. The probable results could provide necessary information to promote new diagnostic methods and potentially improve the health of the pediatric community through public health policies, specific programs, and coordination with other sectors.

## 2. Materials and Methods

### 2.1. Design and Population

This is an observational study based on information obtained from the medical records of all children aged 0–14 who were evaluated for diverse reasons in the outpatient area at the Good Hope Care Center of the district of Villa María del Triunfo, Lima, Peru, between 1 January 2021, and 31 December 2022.

Villa María del Triunfo is a community with approximately 500,000 inhabitants, according to the 2017 census [20]. The population presents social vulnerability and has inadequate health resources since it lacks adequate infrastructure and materials/supplies, and has insufficient human resources to provide quality health care by accepted norms and standards [21]. To add to what was previously mentioned, it has a poverty rate above the average in the city of Lima; 48% of its population is located in sectors D and E (categorized as low income). Even more so, the geographical configuration of the district’s hills makes the execution of sewage works difficult. The population is made up of 19% children, 10% adolescents, and 21% young people. And of the total of high school students enrolled, 17% drop out per year. The fertility rate is 2.41 births per woman and the life expectancy at birth is 78 years, being lower than the Lima average and with an HDI of 0.58 [21].

### 2.2. Study Protocol

First, the medical records of the children aged 0–14 years treated during the study period were collected. The inclusion criteria were as follows: each patient had to have records with the necessary anthropometric data (weight, height, body mass index) and complete laboratory results: red blood cells, hemoglobin, platelets, hematocrit, mean corpuscular volume, mean corpuscular hemoglobin, and percentage of eosinophils. In addition, to be included, they had to have results of a copro-parasitological examination obtained by direct visualization of the parasites under a microscope with physiological saline solution and Lugol staining. Those who did not have all of these data were excluded. In addition, cases that presented more than one type of intestinal parasite on stool examination were excluded and were considered parasitic co-infection.

### 2.3. Definition of Variables

The participants’ demographic information, anthropometric characteristics, and laboratory variables were analyzed. The demographic characteristics were sex, age, and age group (under 2 years, between 2 and 5 years, and over 5 years). Anthropometric characteristics included weight, height, and body mass index (BMI), calculated with the Quetelet formula ([BMI = weight (kg)/height (m)^2^]). The WHO growth curves Weight/Age, Height/Age were calculated using standard deviations, and BMI/Age (not calculated in patients under 2 years of age) was calculated using the Z score. The following laboratory variables were analyzed: red blood cells (by millions), hemoglobin (g/L), platelets (by thousands), hematocrit (%), mean corpuscular volume (MCV), mean corpuscular hemoglobin (MCH), and percentage of eosinophils.

### 2.4. Data Management

The information was obtained with authorized access to the institutional medical records database after the evaluation and approval of the research protocol by the institution’s administrative committee. Patients were identified with randomly generated numerical codes to preserve the confidentiality of the subjects, so data could not be retroactively associated with any patient. Next, the data were transferred to an Excel spreadsheet to verify inconsistencies, duplicates, or errors, guaranteeing the quality of the information for the rest of the variables of interest and then the statistical analysis. The confidentiality of the subjects of this research was respected.

### 2.5. Statistical Analysis

Data analysis was performed with the R language version 4.0.2. Categorical and numerical variables were described as frequencies (%) or medians (interquartile range). For comparisons, chi-squared or Mann–Whitney U was used after the normality assessment (Kolmogorov–Smirnov). Poisson regression models were used to identify the strength of association of each independent variable, estimating adjusted prevalence ratios (aPRs) and 95% confidence intervals (95%CIs). These models were constructed from significant multivariate stepwise associations of Poisson regression with robust variance for each dependent variable (parasites and types) until a final model with bidirectional selection was obtained.

The diagnostic association was evaluated using ROC curves, calculating areas under the curve (AUCs) and 95%CIs. The optimal cut-off points were estimated with the Youden index. Values of *p* < 0.05 were considered statistically significant. The AUC classification values on the ROC curve were no association (<0.50), poor (0.51–0.69), good (0.70–0.79), very good (0.80–0.99), excellent (>0.90) [22].

### 2.6. Ethical Considerations

The Ethics Committee reviewed and approved the study protocol, complying with the research ethical standards. The data were extracted after authorization from the health institution in October 2022. Since the data analyzed were obtained retrospectively from an existing database, the Committee exonerated the researchers from the need to obtain informed consent from individual participants.

## 3. Results

In total, 1894 medical records of patients, of which 951 (50.2%) were girls and 943 (49.8%) were boys, were used in the analysis. It was found that for the male sex (aPR 1.23), the age (aPR 1.06), weight (aPR 0.99), the age group of 2–5 years (aPR 4.54) and >5 years (aPRa 4.76), percentage of eosinophils (aPR 1.02 for every 1% increase), absolute count of eosinophils (aPR 1.17 for every 1% increase), and height/age with −5 SD (aPR 1.33) were associated with a greater risk of parasitosis (Table 1).

**Figure 1:**
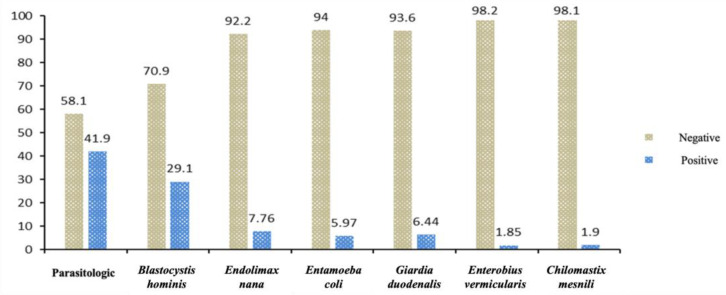
Distribution of positive and negative cases in the parasitological examination of children at the health center.

The prevalence of parasitosis was 41.9%, with the most frequent parasites found being *Blastocystis hominis* (29.1%), *Endolimax nana* (7.76%), *Entamoeba coli* (5.97%), *Giardia duodenalis* (6.44%), and *Enterobius vermicularis* (1.85%) (Figure 1).

The individual analysis for each type of parasite revealed that for *Blastocystis hominis*, there was an association with the male sex (aPR 1.18), age > 2 years (aPR > 12.03), and eosinophilia (aPR 1.02); for *Endolimax nana*, there was an association with eosinophilia (aPR 1.06), an increase in MCH and MCV (aPR 1.19 and 1.06, respectively), and height/age with −5 SD (aPR 2.55); for *Enterobius vermicularis*, there was association with BMI (aPR 0.87), hematocrit (aPR 0.85), and hemoglobin (aPR 0.68); and finally, for *Entamoeba coli* and *Chilomastix mesnili,* there was an association with eosinophilia (aPR 1.05 and 1.06, respectively) and, for the latter, there was an association with height/age with −5 SD (aPR 1.36) (Table 2).

**Figure 2:**
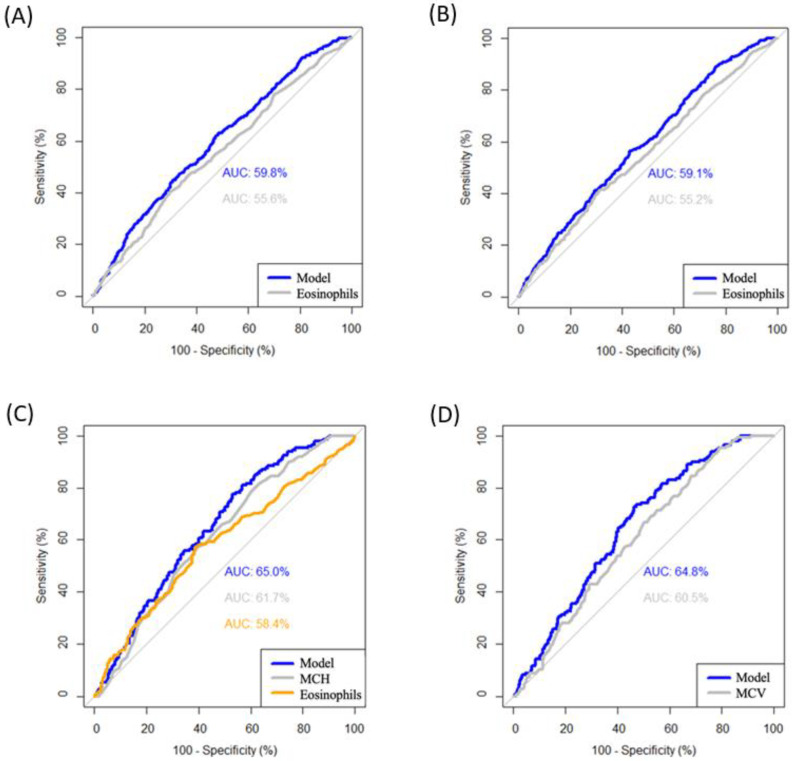
ROC curves of the diagnostic value of the different models associated with the parasites. (**A**) Model for diagnosis of parasites and only with eosinophils (cut-off point: >6%). (**B**) Model for diagnosis of *Blastocystis hominis* and only with eosinophils (cut-off point: >6%). (**C**) Model 1 for diagnosis of *Endolimax nana* and only with eosinophils (cut-off point: >5%) or MCH (cut-off point: >27 pg). (**D**) Model 2 for diagnosis of *Endolimax nana* and only with eosinophils (cut-off point: >5%) or MCV (cut-off point: >82 fl). MCH: mean corpuscular hemoglobin and MCV: mean corpuscular volume.

The Poisson regression models generated to evaluate the diagnostic value of the variables in the study with parasitosis in general and with specific parasites presented the following values: the general model showed a diagnostic value of 59.8% for eosinophilia to detect any parasitosis in male patients over 2 years of age (>6%); without considering age (>6%) the diagnostic value was 55.6% (Figure 2A). Likewise, a diagnostic value of 59.1% was found for eosinophilia to detect *Blastocystis hominis* in male patients older than 2 years (>6%) and of 55.2% without considering age (Figure 2B). On the other hand, the diagnostic value was 65% for Height/Age < −5 SD, eosinophilia >5%, and MCH > 27 pg to detect *Endolimax nana*; 61.7% only for high MCH; and 58.4% only for eosinophilia (Figure 2C). Additionally, a diagnostic value of 64.8% was found in the same population group for MCV > 82 fl instead of MCH, and 60.4% only for high MCV (Figure 2D).

**Figure 3:**
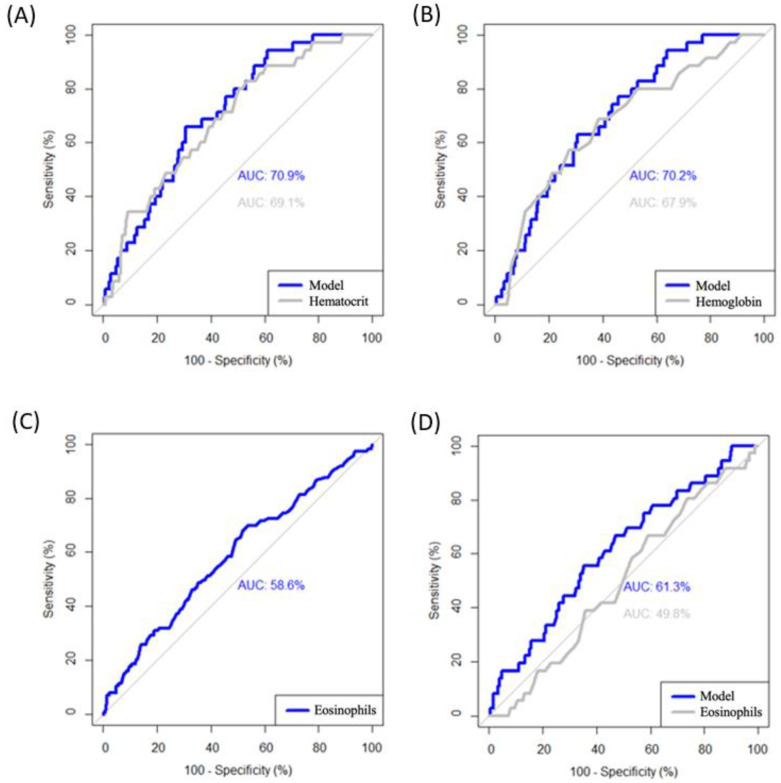
ROC curves of the diagnostic value of the different models associated with the parasites. (**A**) Model for diagnosis of *Enterobius vermicularis* and only with hematocrit (cut-off point: >29.8%) and BMI (cut-off point: <12). (**B**) Model for diagnosis of *Enterobius vermicularis* and only with hemoglobin (cut-off point: <10.6 g/L) and BMI (cut-off point: <12). (**C**) Model for diagnosis of *Entamoeba coli* (cut-off point: >3.7%). (**D**) Model for diagnosis of *Chilomastix mesnili* and only with eosinophils (cut-off point: >5.1%).

Finally, the model yielded diagnostic values of 70.9% and 69.1% for high hematocrit plus BMI < 12 and only high hematocrit, respectively, to detect *Enterobius vermicularis* (Figure 3A); of 70.2% and 67.9% for elevated hemoglobin plus BMI < 12 and only elevated hemoglobin, respectively, to detect *Enterobius vermicularis* (Figure 3B); of 58.6% for elevated eosinophilia to suggest *Entamoeba coli* (Figure 3C); and of 61.3% and 49.8% for growth retardation plus eosinophilia and with eosinophilia alone, respectively, to suggest *Chilomastix mesnili* (Figure 3D).

## 4. Discussion

This study showed a high prevalence (41.9%) of intestinal parasites, mainly due to non-pathogenic species such as *Blastocystis hominis*, *Endolimax nana*, and *Entamoeba coli*. Although these species do not cause disease directly, they share transmission routes with pathogenic protozoa such as *E. histolytica* and *G. duodenalis*. Emerging research has suggested that some of these traditionally non-pathogenic species may be linked to clinical conditions. For example, *Blastocystis hominis*, an opportunistic parasite present in 30–60% of healthy individuals in developing countries, has been associated with Irritable Bowel Syndrome (IBS), Inflammatory Bowel Disease (IBD), and symptoms such as watery diarrhea, abdominal pain, bloating, loss of appetite, and constipation, and, in immunocompromised patients, it may play a pathogenic role [23]. Moreover, the presence of non-pathogenic species can serve as an indicator of the environmental conditions that individuals are exposed to. These commensal species have epidemiological implications since it reflects problems in basic sanitation, sewage network, water quality, and the hygienic habits of school children [24].

Ñacari Sulca et al. reported in 2021 a higher prevalence of *G.* (15.8%), *T. trichiura* eggs (44.7%), and *A. lumbricoides* eggs (39.5%) [24], which contradictorily were not detected in this study. Similarly, De la Cruz et al. found a prevalence of *G. duodenalis* of 10.28% in a similar population in another region of Metropolitan Lima [25,26]. The notably lower prevalence of *G. duodenalis* observed in our study compared to the findings of the previous studies mentioned could be attributed to our study population’s proximity to the medical center in the neighborhood. The medical center’s public health interventions, education programs, and increased awareness about hygiene practices may have contributed to the lower transmission rates of giardiasis. Furthermore, although these studies were conducted within the Villa María del Triunfo district, the vast population can create virtual sub-zones with different water sources or varying water treatment processes. Consequently, the water quality and potential for giardiasis transmission may differ across these sub-zones, potentially explaining the discrepancies in *G. duodenalis* prevalence observed in our study compared to the others.

The result of a 1.85% prevalence of *Enterobius vermicularis* in this study contrasts with a previous study in the same region in 2018, where an 18% prevalence was found in children from a mother–child center. This study concluded that using water in tanks to prepare food, a poor hand-washing habit, and infrequent toileting of children were factors associated with *Enterobius vermicularis* parasitosis. In addition, the incidence of *Enterobius vermicularis* was associated with children’s hand washing at the beginning and end of meals in the institution [27].

It was also demonstrated that children over 2 years of age have more than 4 times the probability of having parasitosis compared to those under 2 years of age, which indicates a greater need for care, monitoring, and treatment in this age group.

Likewise, a significant relationship was found between *Enterobius vermicularis*, BMI, hematocrit, and hemoglobin. Specifically, for each point decrease of hemoglobin, the prevalence of *Enterobius vermicularis* increased by 32% (aPR 0.68), and for each point increase in BMI, the prevalence was reduced by 13% (aPR 0.87). These findings agree with previous studies that show the relationship between anemia and *Enterobius vermicularis* parasitosis in children of the same age group in nearby regions and other countries [14,28]. Although it is known that childhood anemia can lead to acute and chronic malnutrition, in this population, there was no significant association between anemia and *Enterobius vermicularis* with indicators of chronic and acute malnutrition (height/age, weight/age). This finding is consistent with the study by Abraham Degarege et al. in 1205 children, where despite the association between decreased hemoglobin and *Enterobius vermicularis*, there was no association with a reduction in Weight/Height and BMI [29]. However, other studies in different countries have found a significant relationship between *Enterobius vermicularis* and a decrease in the Weight/Age Z-score [14].

Despite the scarcity of studies to analyze the diagnostic association of parasitosis using anthropometric data and blood count, a study by Musa Zorlu et al. in children with a false positive diagnosis of acute appendicitis due to *Enterobius vermicularis* parasitosis was found. These parasites can mimic the clinical presentation of acute appendicitis due to their proximity to the cecum. Therefore, the authors of said study evaluated diagnostic models using blood count data in order to reduce unnecessary surgeries. Its most promising model, based on eosinophilia, presented an association of 65%, which is considered poor [15]. Likewise, other variables associated with *Enterobius vermicularis*, such as biochemical values, could have been investigated. Ahmed Akil et al. found a significant association between *Enterobius vermicularis* infection and deficiency of total serum proteins and serum iron in a group of 505 school children aged 3-10 years [18].

Parasites can induce various hematological alterations in the host, including anemia, leukocytosis, eosinophilia, lymphocytosis, and thrombocytopenia, as well as hematological and hematopoietic disorders. The severity of these alterations depends on factors such as the type of parasite, the degree of parasitemia, and the patient’s immune status [30]. In our results, children with parasitosis did not show altered Hb levels; however, they showed a slight increase in eosinophil levels above the normal range. On the other hand, most of them presented moderate and severe malnutrition. Previous studies have associated the presence of parasitosis with a decrease in body mass index [31,32]. These hematological and nutritional alterations are common in parasitic infections. Therefore, the detection of this condition could serve as an indication of parasitosis, even without confirmation by parasitological examination. Predictive models that integrate these variables, such as the *Enterobius vermicularis* diagnostic model, could support parasitosis diagnosis.

### Limitations

The main limitations of this study were the absence of an analysis of other parasites frequently associated with anemia, eosinophilia, and anthropometric alterations, such as *Ascaris lumbricoides* and *Trichuris trichiura*, as well as patients with multi-parasitic infections. Some undiagnosed coinfections that could only be diagnosed through serological tests were not studied, mainly due to the lack of availability of those tests in that area. More relevant demographic data, such as family educational level and clinical data from the history and physical examination, must be included. This information could have allowed us to develop other, more precise diagnostic value models for parasitosis and thus increase the transferability to clinical practice.

Another limitation is including the entire population attending the medical center during the study period rather than selecting a sample. While this approach ensured comprehensive data collection from all available cases, it may limit the generalizability of our findings. Similarly, the variability in physiological changes caused by different types of parasites complicates the development of a unique diagnostic model for parasitosis. Further research is needed to validate and enhance the diagnostic accuracy of our proposed models by using control groups, assessing their value in relation to parasitological exams, and exploring other predictive models incorporating additional factors such as environmental conditions, nutritional status, or more advanced statistical techniques like machine learning. Such studies could lay the foundations for new diagnostic strategies, health sector regulations, and programs focused on the early detection of parasitic infections in vulnerable populations, potentially accelerating diagnosis and treatment, thus mitigating their negative impact on child growth and development.

## 5. Conclusions

This study highlights the need to implement rapid, affordable, and accessible diagnostic methods for the detection of parasitosis in low-resource settings, where copro-parasitological exams are often not readily available. The prevalence of parasitosis in the studied population differed from other previous regional and socioeconomic studies. The set of BMI < 12 and hematocrit > 29.8% and the set of BMI < 12 and hemoglobin < 10.6 g/L may form two diagnostic models with 70.9% and 70.2% diagnostic association, respectively, for *Enterobius vermicularis* parasitosis, which could be used for the diagnosis of parasitosis in high-risk zones. Further studies are needed to establish this association, using a combination of statistical and clinical criteria to ensure greater relevance. The possibility of annual empirical treatment should also be considered. While empirical treatment may help control parasitic infections in high-risk areas, it carries the potential drawback of unnecessary drug administration, the development of drug resistance, and the masking of underlying health conditions. Therefore, carefully evaluating local circumstances and weighing the benefits against the risks is crucial before implementing such an approach. More studies are required to seek new diagnostic strategies and treatment, thus mitigating their negative impact on child growth and development from those areas.

## Figures and Tables

**Table 1:** General characteristics and comparison of variables between parasitic and non-parasitic groups in children treated at the health center.

Variables	Total (n = 1894)	Parasitosis		*p*	aPR
		Negative (n = 1101)	Positive (n = 793)		
Sex (%)				<0.001 **	
Girls	951 (50.2%)	596 (62.7%)	355 (37.3%)		Reference
Boys	943 (49.8%)	505 (53.6%)	438 (46.4%)		1.23 ^a^ (1.06–1.31) **
Age (years)	7.00 [5.00–10.0]	7.00 [4.00–10.0]	8.00 [5.00–11.0]	<0.001 **	1.06 ^a^ (1.03–1.09) **
Weight (kg)	24.7 [16.9–38.0]	23.6 [15.4–37.1]	25.8 [18.5–39.0]	<0.001 **	0.99 ^a^ (0.98–0.99) **
Size (m)	1.22 [1.05–1.39]	1.20 [1.00–1.38]	1.24 [1.09–1.40]	<0.001 **	1.16 ^a^ (0.98–1.29)
BMI (kg/m^2^)	17.0 [15.3–19.9]	17.0 [15.4–19.9]	17.0 [15.3–19.8]	0.869	0.99 ^e^ (0.95–1.02)
Red blood cells (millions)	4.53 [4.31–4.77]	4.53 [4.29–4.77]	4.54 [4.32–4.77]	0.469	0.99 ^a^ (0.84–1.17)
Hemoglobin (g/L)	12.4 [11.7–13.0]	12.3 [11.6–13.0]	12.5 [11.7–13.1]	0.005 **	0.91 ^e^ (0.22–3.27)
Platelets (thousands)	341 [295–392]	343 [298–393]	338 [292–389]	0.171	1 ^b^ (0.99–1.01)
Hematocrit (%)	37.2 [35.0–39.3]	37.0 [34.9–39.3]	37.4 [35.4–39.4]	0.012 **	1.00 ^d^ (0.98–1.02)
MCV (fl)	82.1 [79.5–84.7]	82.1 [79.3–84.7]	82.3 [80.0–84.8]	0.027 *	1.01 ^c^ (0.99–1.02)
MCH (pg)	27.4 [26.4–28.2]	27.3 [26.3–28.2]	27.4 [26.5–28.3]	0.006 **	1.02 ^b^ (0.98–1.05)
Eosinophils (%)	4.10 [2.40–7.10]	3.90 [2.20–6.70]	4.60 [2.50–8.00]	<0.001 **	1.02 ^a^ (1.01–1.03) **
Absolute eosinophils	0.30 [0.16–0.54]	0.28 [0.16–0.50]	0.33 [0.18–0.58]	<0.001 **	1.17 ^b^ (1.04–1.31) **
Age group (%)				<0.001 **	
<2 years	61 (3.22%)	57 (93.4%)	4 (6.56%)		Reference
2 to 5 years	536 (28.3%)	331 (61.8%)	205 (38.2%)		4.54 ^b^ (2.23–10.99) **
>5 years	1297 (68.5%)	713 (55.0%)	584 (45.0%)		4.76 ^b^ (2.41–11.31) **
Weight/age (%)				<0.001 **	
−3 SD	435 (23.0%)	235 (54.0%)	200 (46.0%)		Reference
−2 SD	513 (27.1%)	347 (67.6%)	166 (32.4%)		0.99 ^c^ (0.81–1.21)
−1 SD	247 (13.0%)	127 (51.4%)	120 (48.6%)		0.99 ^c^ (0.81–1.21)
0 SD	198 (10.5%)	115 (58.1%)	83 (41.9%)		0.99 ^c^ (0.81–1.21)
2 SD	206 (10.9%)	114 (55.3%)	92 (44.7%)		0.99 ^c^ (0.81–1.21)
1 SD	203 (10.7%)	106 (52.2%)	97 (47.8%)		0.82 ^c^ (0.52–1.28)
3 SD	61 (3.22%)	36 (59.0%)	25 (41.0%)		0.82 ^c^ (0.52–1.28)
4 SD	31 (1.64%)	21 (67.7%)	10 (32.3%)		0.82 ^c^ (0.52–1.28)
Height/age (%)				<0.001 **	
−3 SD	447 (23.6%)	310 (69.4%)	137 (30.6%)		Reference
−5 SD	1447 (76.4%)	791 (54.7%)	656 (45.3%)		1.32 ^b^ (1.05–1.65) *
BMI/age Z score (%)				<0.001 **	–
−2 SD	447 (23.6%)	310 (69.4%)	137 (30.6%)		Reference
−3 SD	1447 (76.4%)	791 (54.7%)	656 (45.3%)		1.1 ^d^ (0.89–1.05)

Variables are presented as median [interquartile range] or absolute and relative frequency (%). aPR, adjusted prevalence ratio; * *p <* 0.05, ** *p <* 0.01, statistically significant by Mann–Whitney U, chi-square, or Poisson regression. Poisson regression models present a multivariable approach to all variables in each model; ^a^ Sex, Age in years, Weight, Size, Red blood cells, % of Eosinophils; ^b^ Sex, Age group, Height/age, MCH, Absolute eosinophils, Platelets; ^c^ Sex, Age in years, MCV, Weight/age; ^d^ Sex, Age in years, Hematocrit, BMI/age Z score; ^e^ Sex, Age in years, BMI, Hemoglobin. MCV: mean corpuscular volume, MCH: mean corpuscular hemoglobin, BMI: body mass index, SDs: standard deviations.

**Table 2:** Poisson regression models of different variables associated with diagnosis of parasitosis.

Variables	*Blastocystis hominis*		*Endolimax nana*		*Enterobius vermicularis*		*Entamoeba coli*		*Chilomastix mesnili*	
	aPR	(95%CI)	aPR	(95%CI)	aPR	(95%CI)	aPR	(95%CI)	aPR	(95%CI)
Sex										
Girls		Reference	†		†		†		†	
Boys	1.18	(1.03–1.36) *	†		†		†		†	
Age group										
<2 years		Reference	†		†		†		†	
2 to 5 years	12.03	(3.22–116.36) **	†		†		†		†	
>5 years	13.6	(3.53–133) **	†		†		†		†	
Eosinophils %	1.02	(1.01–1.03) *	1.04	(1.01–1.06) **	†		1.05	(1.02–1.08) **	1.06	(1.00–1.11) *
Height/age										
−3 SD	†			Reference	†		†			Reference
−5 SD	†		2.55	(1.48–4.75) **	†		†		1.36	(0.61–3.59) *
MCH	†		1.19	(1.06–1.33) **	†		†		†	
MCV	†		1.06	(1.01–1.10) *	†		†		†	
BMI	†		†		0.87	(0.78–0.97) *	†		†	
Hematocrit	†		†		0.85	(0.77–0.95) **	†		†	
Hemoglobin	†		†		0.68	(0.51–0.92) *	†		†	

For: adjusted prevalence ratio. * *p* < 0.05, ** *p* < 0.01, statistically significant by Poisson regression. Poisson regression models present a multivariable approach to all variables in the model. †: the variable does not enter in the model analysis.

## Data Availability

The dataset is contained as a Supplementary Material (Supplementary Material S1).

## Supplementary Materials

The following supporting information can be downloaded at: https://www.mdpi.com/article/10.3390/children11121416/s1.
